# A regulatory network modeled from wild-type gene expression data guides functional predictions in *Caenorhabditis elegans* development

**DOI:** 10.1186/1752-0509-6-77

**Published:** 2012-06-26

**Authors:** Brandilyn Stigler, Helen M Chamberlin

**Affiliations:** 1 Department of Mathematics, Southern Methodist University, Dallas, TX 75275, USA; 2 Department of Molecular Genetics, Ohio State University, Columbus, OH, 43210, USA

## Abstract

**Background:**

Complex gene regulatory networks underlie many cellular and developmental processes. While a variety of experimental approaches can be used to discover how genes interact, few biological systems have been systematically evaluated to the extent required for an experimental definition of the underlying network. Therefore, the development of computational methods that can use limited experimental data to define and model a gene regulatory network would provide a useful tool to evaluate many important but incompletely understood biological processes. Such methods can assist in extracting all relevant information from data that are available, identify unexpected regulatory relationships and prioritize future experiments.

**Results:**

To facilitate the analysis of gene regulatory networks, we have developed a computational modeling pipeline method that complements traditional evaluation of experimental data. For a proof-of-concept example, we have focused on the gene regulatory network in the nematode *C. elegans* that mediates the developmental choice between mesodermal (muscle) and ectodermal (skin) cell fates in the embryonic C lineage. We have used gene expression data to build two models: a knowledge-driven model based on gene expression changes following gene perturbation experiments, and a data-driven mathematical model derived from time-course gene expression data recovered from wild-type animals. We show that both models can identify a rich set of network gene interactions. Importantly, the mathematical model built only from wild-type data can predict interactions demonstrated by the perturbation experiments better than chance, and better than an existing knowledge-driven model built from the same data set. The mathematical model also provides new biological insight, including a dissection of zygotic from maternal functions of a key transcriptional regulator, PAL-1, and identification of non-redundant activities of the T-box genes *tbx-8* and *tbx-9*.

**Conclusions:**

This work provides a strong example for a mathematical modeling approach that solely uses wild-type data to predict an underlying gene regulatory network. The modeling approach complements traditional methods of data analysis, suggesting non-intuitive network relationships and guiding future experiments.

## Background

Production of the diverse cell types that make up a multicellular organism is a highly complex and interconnected process. Although at the cellular level many developmental decisions can be broken down to a simple choice between two outcomes, it is clear that there are multiple regulatory inputs into that decision (reviewed by [1]). While a variety of genetic and genomic methods can be used to dissect the regulatory inputs into developmental cell fate decisions, large scale experimental analyses are limited by time and expense. These practical constraints argue for the development of computational methods that maximize extraction of biologically relevant information from the available data, as well as the development of predictive models to prioritize experiments for future testing.

Mathematical approaches to modeling complex behavior can generally be categorized as either data-driven or knowledge-driven. Knowledge-driven models are constructed by assembling all known features of a biological system into the model. Knowledge-driven models can be considered “bottom up” models, and they reflect a methodology that has been highly effective in the physical sciences. Although knowledge-driven models are common in mathematical biology [2-4], it is often the case that there is not sufficient information about the biological system to completely define the model. In contrast, data-driven models are built from experimental data, often in the absence of known features. These models are “top down,” in that they use observations from the biological system to infer, or reverse engineer, the model. Data-driven models can always be built given an appropriate data set; the problem lies in the fact that there are typically hundreds to thousands of possible models for a given data set and so model selection techniques must be employed.

There are many reverse-engineering methods available to select preferred network models from among the possible set [5-8]. However, these methods often rely on certain types of data, such as gene expression data resulting from the perturbation of other genes in the network. Relatively few methods perform well for data recovered only from the wild-type condition and fewer still correctly provide directional information, such that the regulatory relationships among genes are predicted [9]. However, limiting the practice of model-building to experimental frameworks in which systematic perturbations have been performed limits the range of biological systems that are readily available to mathematical modeling, especially for large regulatory networks. Thus we set out to develop a mathematical modeling strategy that utilizes data collected only from the wild-type condition, yet enriches for regulatory interactions observed in perturbation experiments.

**Figure 1:**
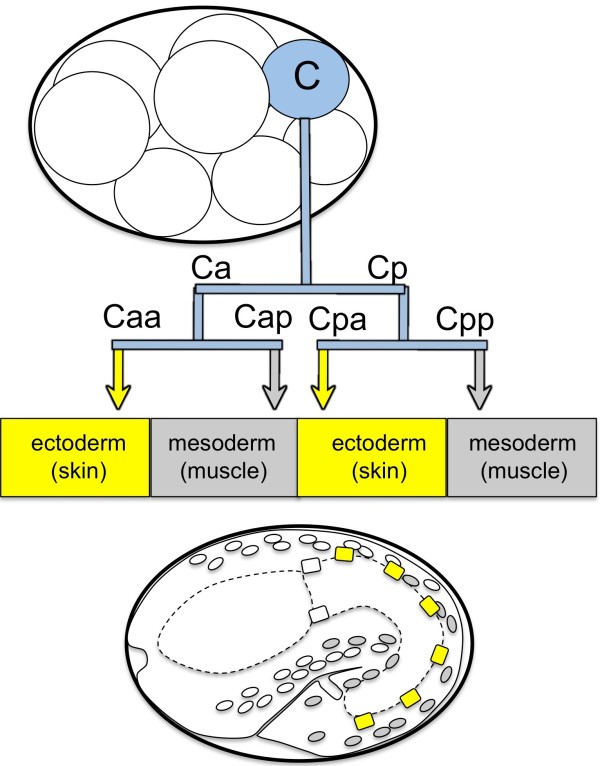
**The C lineage produces ectodermal and mesodermal cells in the*C. elegans*embryo.** The C cell is an embryonic “founder” cell in the *C. elegans* embryo that is born after three rounds of cell division. It divides to produce cells that contribute to the ectoderm (skin) and mesoderm (body wall muscle) of the embryo, which are highlighted in the bottom diagram of a 1.5 fold embryo.

This work uses data from experiments on the nematode *C. elegans*, an experimental model used to study the genetics and cell biology of a variety of developmental processes. During *C. elegans* embryonic development, the fate of different lineage precursor cells is established, making each precursor different from the others ([10], reviewed in [11]). The development of one cell, termed the C cell, is dependent on the maternally-supplied homeodomain transcription factor PAL-1 ([12]; Figure1). The C cell is precursor to two distinct cell types: mesoderm (muscle) and ectoderm (skin). Following specification of the C cell type by PAL-1, interaction among a number of PAL-1-dependent genes results in the decision of a cell to differentiate as mesoderm or ectoderm. Baugh *et al.*[13] developed a preliminary, biological model for the C lineage regulatory network based on a developmental time course of gene transcript abundance data from animals wild type for all genes in the network. This model was then tested and refined by systematic gene disruption and gene interaction experiments of Yanai *et al.*[14]. This combined set of wild-type descriptions and perturbation experiments provides data sets that allow for the building and testing of mathematical models for this developmental process. Consequently, we have selected this experimental system as a proof-of-concept example to compare the performance of data-driven mathematical models with that of knowledge-driven biological models.

Here, we present a mathematical approach that uses two distinct methods in series to model this developmental network solely from wild-type gene transcript abundance time-course data. To test the mathematical model, we build a corresponding knowledge-driven network that is comprehensive in the sense that it incorporates interactions for which there are multiple sources of supporting experimental evidence. We find that the mathematical model predicts regulatory relationships present in the biologically-derived network with a high degree of accuracy, and that it predicts more features of the biological network than does a knowledge-driven model derived from the same data set. The results argue that model-assisted evaluation of experimental data can identify new regulatory relationships not suggested by existing scientific knowledge, and can guide future experimental inquiry.

## Results

### Overview of modeling and model validation framework

The research framework relies on two data sets that represent different states of knowledge for the same gene regulatory network. The gene regulatory network includes a set of genes important for the development of the *C. elegans* embryonic C cell lineage, a cell that gives rise to two distinct cell types: mesoderm and ectoderm [10]. The C lineage is dependent on the transcription factor PAL-1, and a number of genes have been identified that influence how cells in the lineage choose between the two possible cell type outcomes [12,13]. The test data set is based largely on results of Yanai *et al.*[14], who completed gene perturbation experiments followed by transcript abundance analysis for all genes in the network, and yeast one hybrid (DNA binding) analysis for all transcription factors and the upstream sequences for each gene in the network. These data (along with additional data curated from the literature, see Methods) are used to develop the Gold Standard Network (GSN). The source data set for the Mathematically Inferred Model (MIM) is provided in [13]. It includes time-course analysis of gene transcript abundance in essentially wild-type animals. In addition, we have formalized the knowledge-driven model proposed by Baugh *et al.* (Figure 9 of [13]) as the Wild Type Model (WTM), for comparison to the MIM. Consequently, both the mathematical model (MIM) and the knowledge-driven biological model (WTM) are derived from the same data set and reflect a comparable state of knowledge of the gene regulatory network. These two models are then compared to the Gold Standard Network derived from the test data set. To select the genes to include in the network, we used all of the genes in the network proposed by Baugh *et al.*[13], plus *lin-26*. *lin-26* had been previously identified as a critical factor for development of epidermal cell types [15-17]. This approach of separating the model-building data from the model-testing data provides a rigorous test of our mathematical modeling strategies using data that are pre-existing in the literature.

We will use the word “module” to refer to groups of genes, following the groupings of Yanai *et al.*[14], with additions from Baugh *et al.*[13]. The specific groups are “initiation” for *pal-1, tbx-8,* and *tbx-9*; “ectoderm” for *elt-1, elt-3, lin-26,* and *nhr-25*; “mesoderm” for *hlh-1, hnd-1,* and *unc-120*; “mixed” for *nob-1, scrt-1,* and *vab-7*; and “other” for *cwn-1* and *mab-21*. In all figures we have color coded the nodes according the module: blue for initiation, yellow for ectoderm, gray for mesoderm, brown for mixed, and green for other.

### The gold standard network is a comprehensive knowledge-driven model of the gene regulatory network controlled by PAL-1

In order to assess the performance of a model, a representation of the "truth", or a so-called gold standard network, is required. A network is defined as a *gold standard* if it is used to validate the performance of a method or a model; in essence it is considered to be the sought-after solution [18,19]. While it is common to construct a gold standard from a synthetic or a simulated network, we aim to assess the predictive power of two types of models (*i.e.*, data-driven versus knowledge-driven) in the presence of future knowledge. Therefore our gold standard, constructed from interactions obtained from experiments subsequent to those in [13], is intended to be a comprehensive model of the regulatory network controlled by PAL-1.

**Figure 2:**
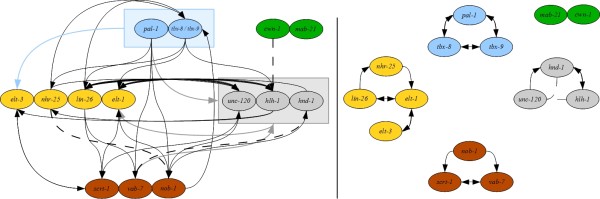
**The Gold Standard Network (GSN) derived from the literature.** The GSN is represented as a pair of mixed graphs, where solid lines with arrows indicate directed interactions and dashed lines without decorations indicate undirected interactions: the left graph depicts interactions between modules, and the right graph depicts interactions within modules. Since there is no difference between incoming and outgoing edges for the genes *tbx-8* and *tbx-9*, they are represented as a single node in the between-groups graphs to minimize the number of edges displayed. The colors of the nodes, maintained throughout, correspond to their associated module: blue for initiation, yellow for ectoderm, gray for mesoderm, brown for mixed, and green for other.

The primary source of data for our gold standard network, which we label GSN, regulating cell fate decisions in the *C. elegans* C lineage is [14], with additional data curated from the literature (Additional file 1: Table S 1, tabs “Gene interactions” and “Gene interactions – refs”). Yanai *et al.* incorporated their results into a knowledge-driven model represented as a set of directed graphs (Figure 4 of [14]), which we refer to as the Experimentally Derived Model (EDM). While this model reflects the data, we have produced an alternative model that is derived from a systematic approach to data interpretation (see Methods). The rationale for production of this alternative model is to demonstrate one way in which scientists lacking specialized expertise in a particular biological system can use existing data to build a knowledge-driven model, and derive testable hypotheses from that model. We define this as the GSN. Due to its size, the GSN is represented as a pair of graphs: one showing the interactions between genes within a regulatory module (*e.g.*, ectoderm, mesoderm) and the other showing the interaction between genes in different modules (Figure2). The model includes both directed and undirected edges, depending on the type of experimental data that predict the edge. For consistency, we use the gene (rather than protein) names in all of the models.

**Figure 3:**
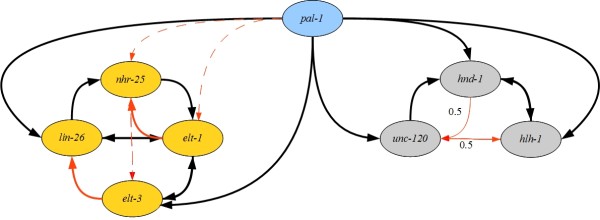
**Comparison of the GSN to the EDM represented as a consensus graph.** Black edges reflect agreement, whereas red edges reflect differences between the two models. Solid thick red edges depict edges in the EDM that are not in the GSN; that is the GSN predicts that there is no interaction between the genes incident to a solid red edge. Dashed thin red edges depict edges in the EDM for which there are no predictions in the GSN; that is, according to the GSN, it is unknown whether the genes incident to a dashed red edge interact. Solid thin red edges annotated with 0.5 depict edges predicted to be directed in the EDM and undirected in the GSN.

To evaluate the GSN, we compared it to the EDM, including only *pal-1* and the genes of the ectoderm and mesoderm modules, to match the choices in [14]. In Figure3 we illustrate the similarities (in black) and differences (in red) between the EDM and the GSN. The GSN shares extensive similarity with the EDM, a result that is not unexpected given that the two draw on the same data sources. Two edges in the ectodermal module (from *elt-1* to *nhr-25*, and *elt-3* to *lin-26*) are included in the EDM, whereas they are predicted to be absent by the GSN. Five additional edges are predicted by the EDM, but the data were insufficient for the GSN to either support the edge, or provide a direction for the edge. We hypothesize that the requirement of two data features to support inclusion of an edge in the GSN will result in a more conservative network than provided by the EDM. Altogether, the GSN is a network that shares similarity with one derived independently by experimental specialists.

One benefit of the GSN is that it incorporates data for all genes in the network, allowing description of features that are not included in the EDM. For example, we observe that the initiation genes *pal-1* and *tbx-8/tbx-9* are distinct in their interactions within the GSN, whereas no such distinction is made in the EDM. In particular, *pal-1* and *tbx-8/tbx-9* have different target gene sets. In addition, *tbx-8/tbx-9* are regulated by other genes in the network (*nhr-25*, *lin-2*6 and *nob-1*), whereas *pal-1* has no in-network regulators. In the GSN all three of the initiation genes (along with others) regulate *elt-3*, whereas in the EDM *pal-1* is the only regulator from the gene set. In the GSN, we see that *pal-1* regulates all mesodermal genes, which matches the predictions in the EDM; however, we also see that the other two initiation genes *tbx-8/tbx-9* also regulate *hlh-1*. Altogether, the GSN includes a greater complexity of interactions for the initiation gene set than does the EDM.

The EDM incorporates data for two *pal-1*-regulated modules: ectoderm and mesoderm. The EDM and GSN exhibit similarities within the ectoderm module, but there are a number of differences for the mesoderm module. The EDM predicts that all three genes regulate each other. The GSN is in agreement between *hnd-1* and *hlh-1*; however there is disagreement around *unc-120*. The difference arises from there not being enough support for the regulatory interactions involving *unc-120*. In fact, the GSN suggests that the regulatory interaction from *hnd-1* to *unc-120* may happen through *nob-1*; that is, there is a directed path of *hnd-1 →* *nob-1 →* *unc-120.* While additional data might modify the interpretation, the more conservative GSN identifies the potential for indirect regulatory relationships.

For the mixed module, the EDM provides no predictions for these genes. While we cannot make a direct comparison, we will highlight some predictions of the GSN. The GSN identifies the mixed gene *vab-7* as a key regulator of the mesoderm module, as only it and *pal-1* regulate all three genes of the module. The GSN also identifies *nob-1* as an important regulator in the whole network. *nob-1* interacts with each module through regulation of *elt-1* (ectoderm), *unc-120* (mesoderm), and *tbx-8/tbx-9* (initiation). *nob-1* also regulates the other genes within the mixed module. In contrast, *scrt-1* exhibits no interactions beyond the mixed module. Thus the GSN identifies the cross-network nature of genes in the mixed module, and also demonstrates functional differences among the mixed module genes.

The authors of the EDM proposed an “expert-inferred” model (*i.e.*, one not strictly based on their data) for the regulation of the ectoderm and mesoderm modules by *pal-1* (see Figure 6 of [14]); we refer to this model as the C lineage model. For completeness’ sake, we compare the GSN to those additional findings. In the C lineage model, the authors predict that the ectoderm gene *elt-1* regulates the mesoderm module; the GSN narrows the target set to *unc-120* and *hlh-1*, and includes *lin-26* as another regulator of these two mesoderm genes. Additionally, in the GSN the mesoderm module regulates *elt-1*, which matches the predictions in the C lineage model. Thus the GSN also makes predictions that match expert-driven network features that reflect expert experience, knowledge of the literature, and knowledge of the experimental system.

In building the GSN, we also collected information about which genes are observed to not regulate other genes, referred to as “non-interactions”. Non-interactions are distinct from relationships for which there is insufficient data, and identify prospective cases of network hierarchy or other network constraints. For example, a number of non-interactions were identified between initiation genes and genes from other categories. The genes *elt-1*, *elt-3, hlh-1, unc-120,* and *scrt-1* were found to not regulate any of the initiation genes. Additionally *lin-26, nhr-26, hnd-1, vab-7,* and *nob-1* do not regulate *pal-1*. In the ectoderm module, we observe that *nhr-25* and *elt-3* do not regulate *lin-26; elt-1* does not regulate *nhr-25;* and *lin-26* does not regulate *elt-3*. In the mesoderm module, however, no non-interactions were discovered. These results identify differences in either the interconnectedness or the functional redundancy between the two modules.

To further evaluate the GSN, we evaluated a set of “statistics” from graph theory for which common features have been discovered in modeling gene regulatory networks [20]. In many biological networks, the average path length (average minimal number of edges between any two nodes) is less than four. With an average path length of about three, the GSN is consistent with this prediction. The average out-degree (the number of outgoing edges) is five and the average in-degree of the nodes is five. Consistent with other gene regulatory networks, the GSN has relatively few nodes with an out-degree of greater than half of the network in their target set: *pal-1lin-26*, and *nob-1*. These genes can be considered the network hubs. While there are also relatively few nodes with an in-degree of greater than half of the network (*hlh-1* and *elt-1*), the GSN is unusual for a transcriptional network as it has a relatively large range for the in-degree of the nodes (0–10 in a 15 node network). Overall, the GSN exhibits network features seen in other gene regulatory networks, and serves as a knowledge-based model for C cell lineage development against which other models can be tested.

### A mathematically inferred model of the gene regulatory network controlled by PAL-1

**Figure 4:**
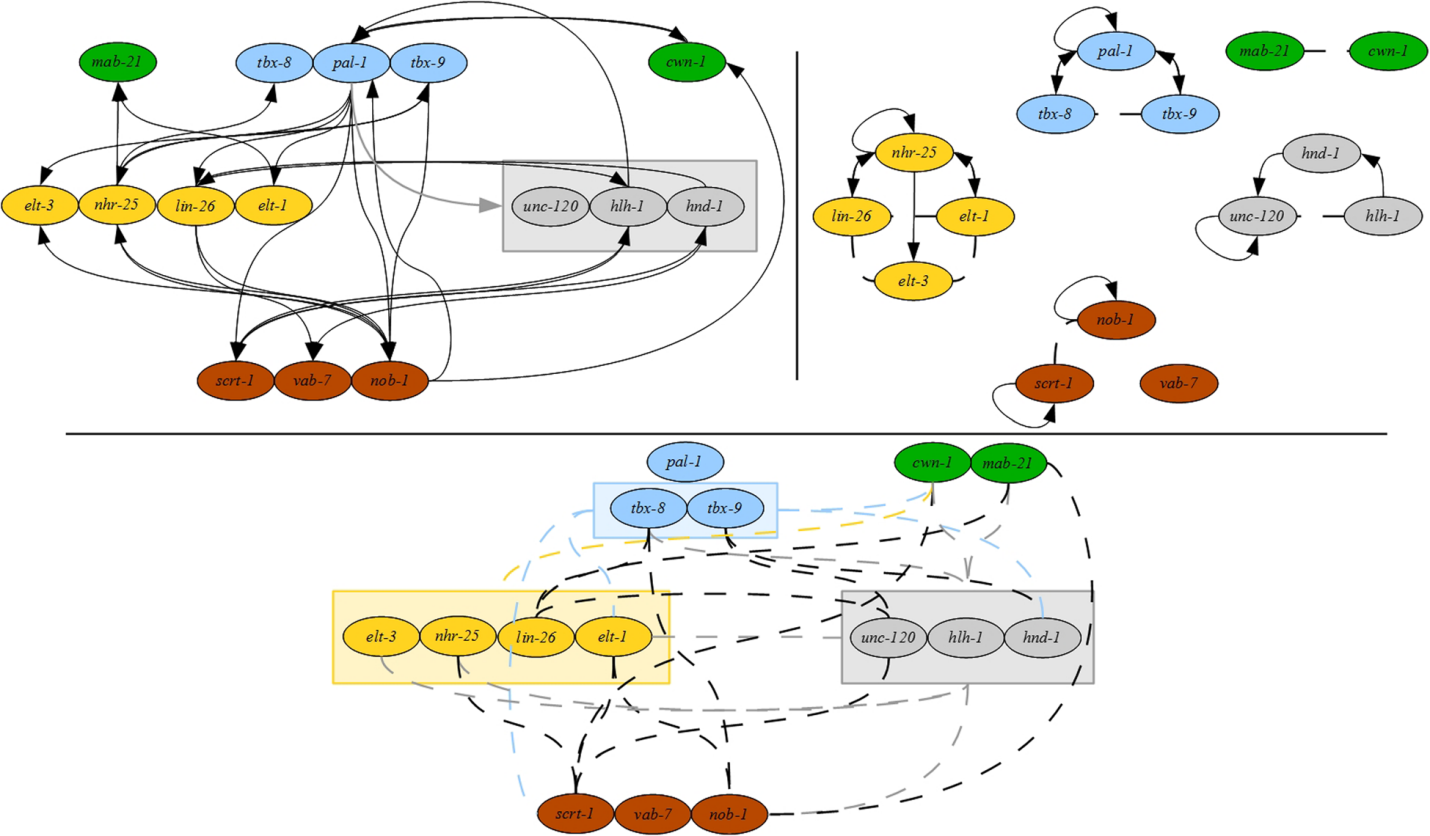
**The Mathematically Inferred Model (MIM) constructed using MSA-COV.** The MIM is represented as a triple of mixed graphs, where solid lines with arrows indicate directed interactions and dashed lines without decorations indicate undirected interactions: the upper left graph depicts directed interactions between modules; the upper right graph depicts interactions within modules; and the bottom graph depicts undirected interactions between modules. A colored edge incident to a colored box indicates that the edge is incident to all genes in the box; edge and box colors are used to assist the reader in determining the ends of edges.

We derived a Mathematically Inferred Model (MIM) for the gene network regulating cell fate decisions in the *C. elegans* C cell lineage using wild-type gene expression time-course data from [13]. The model results from applying two different modeling methods in series. Such approaches have been termed “pipelines,” and they exhibit improved performance over individual methods alone [21]. To utilize the benefits and minimize the weaknesses of methods from different modeling classes, we applied statistical (covariance (COV)) and algebraic (the Minimal Sets Algorithm (MSA)) methods in series (see Methods). Covariance can discover a larger number of gene relationships, but does not provide information about the regulatory relationship between genes that covary. MSA, on the other hand, identifies fewer possible relationships, but it predicts directional edges since it incorporates the observed gene expression changes from one time point to the next. Consequently, the model includes both directed and undirected edges, reflecting the types of edges predicted by the different methods. The MIM is represented as a set of three figures: one showing the interactions between genes within a regulatory module, one showing directed interactions between genes in different modules, and one showing undirected interactions between genes in different modules (Figure4).

There are two principal ways to combine the methods used in the MIM: COV followed by MSA, denoted COV-MSA, and vice versa. Intuitively it seems natural to first allow COV to decide which genes interact and then to let MSA determine the direction of the interaction; however, either order is technically feasible. We built a model using both pipeline orders, compared them using the assessment methods described below, and found that they yield comparable results (see Methods). These findings suggest that, at least in this case, the order of modeling methods within the pipeline does not drastically impact the performance of the model. The MIM of Figure4 results from application of MSA-COV, as this model is modestly better than that built from the reverse order. The graphs from application of COV-MSA are shown in Figures S1 and S2 in Additional file 2.

**Figure 5:**
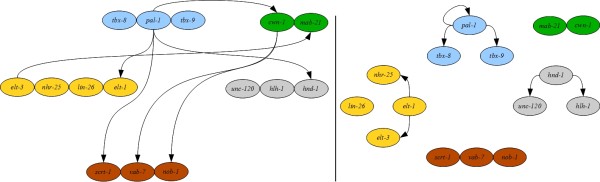
**The Wild Type Model (WTM), derived from Baugh*et al.*[**[13]**].** The WTM is represented as a pair of directed graphs, where solid lines with arrows indicate directed interactions: the left graph depicts interactions between modules, and the right graph depicts interactions within modules.

**Figure 6:**
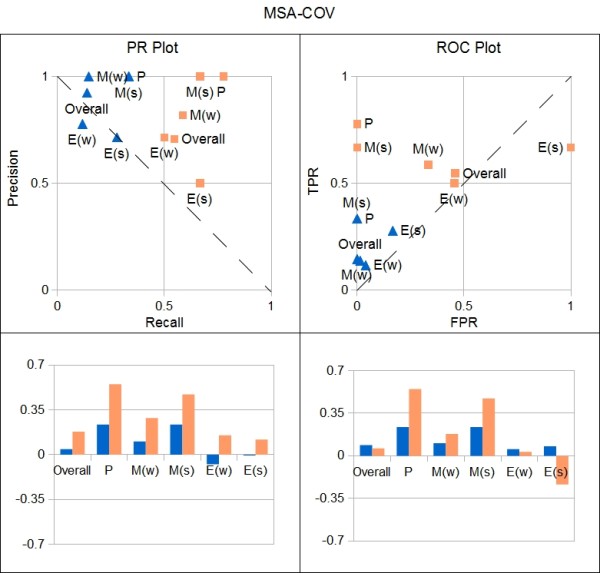
**Performance of the MIM using MSA-COV.** In the upper left is a precision-recall (PR) plot and in the upper right is a receiver operating characteristic (ROC) plot. Blue triangles and orange squares represent data points for the WTM and the MIM, respectively. The labels for the data points are as follows: E = ectoderm module; M = mesoderm module; P = targets of PAL-1; Overall = entire model. A parenthetical “s” for “*modulesubnetwork*” indicates performance of the subgraph generated by genes within a module; that is, edges incident to genes outside of the module are not counted. A parenthetical “w” for “*module inwhole network*” indicates performance of the subgraph induced by those edges incident to genes within a module; note that genes outside the module may be included in the subgraph. A good value lies in the upper right-hand corner of the PR graph and in the upper left-hand corner of the ROC graph. In either case, values that lie on the dashed line are considered to be no better than random guesses and values below the line are considered to be poor. In the bottom row are the distances of the points in the upper plots from the dashed lines.

To assess the MIM, we compared it to the GSN, and to the model offered by Baugh *et al.* ([13], their Figure 9). We term this model the Wild Type Model (WTM; Figure5), as it is a knowledge-based model derived from the same wild-type data used for the MIM. As a first comparison, we evaluated the number of predictions in each model that are validated by the GSN. The WTM model makes 39 predictions, which includes true positives, true negatives, false positives, and edges considered to be half-right (see Methods) with 32 or 82% being correct, whereas the MIM makes 88 predictions with 57 or 65% being correct. Thus although the WTM achieves a higher percentage of correct predictions, the MIM makes over twice as many predictions, without a comparable loss of correctness. For a more detailed comparison of the models, we used precision-recall (PR) and receiver operating characteristic (ROC) plots (Figure6). In both graphs, points that lie above the dashed line have a stronger predictive value than random. The data used to produce these figures are included in Additional file 1: Table S 1, Tab “Overall.”

We assessed the models’ overall performance, as well as performance on ability to identify targets of PAL-1 and to identify the ectoderm and mesoderm subnetworks. More specifically, we considered the subnetworks generated by the genes within a module, that is the subgraph of edges incident only to genes within a module. We also considered the subnetworks of the modules within the context of the entire network; that is the subgraph induced by those edges incident to genes within a module and possibly incident to genes outside the module. The subnetwork of the first type is denoted by “s” and of the second type, by “w” in Figure6. Note that two nodes are *adjacent* if they share an edge, and an edge is *incident* to a node if the node is an endpoint of the edge.

A global comparison of the models can be obtained by evaluating the distance of the resulting model from one whose predictions are random guesses (see Methods). The MIM has a total distance of 2.8, whereas a best performing model has a total distance of about 8.49. In contrast, the total distance of the WTM from random is 1.3, arguing that the MIM globally has a 111% increase in predictability over the WTM. The predictions that the WTM (blue triangles) makes are correct in the context of the GSN, accounting for high precision (92%) and low FPR (1%); however, the WTM misses many of the interactions in the GSN, which is evident in its low recall (14%). It is due to the low recall value that the predictions of the WTM have a distance of 0.04 and 0.09 from random guesses in the PR and ROC plots, respectively. While the MIM (orange squares) has more misidentified predictions (lower precision at 71% and higher FPR at 46%) than the WTM, it has greater recall (55%). These changes result in a distance of 0.18 and 0.06 from random guesses in the PR and ROC plots, respectively, indicating a 316% increase in PR space and a 29% decrease in ROC space. We see that the MIM correctly identified more targets of PAL-1 (78% recall) than the WTM (33% recall); both models have 100% precision and 0% FPR for the targets of PAL-1. The MIM’s PR and ROC points are a distance of 0.55 from a random guess, compared to the WTM’s points at a distance of 0.24; this value of 0.55 is the largest distance achieved by either model. The MIM also identified more interactions in the mesoderm module (59-67% recall; the range reflects differences between subnetwork “s” vs “w”) than the WTM (15-33% recall). While the WTM has 100% precision and 0% FPR on the mesoderm module, the MIM has 82-100% precision and 0-33% FPR. The significance is that the MIM does not lose much in the way of making mistakes, while it gains much in the way of identifying other interactions, as compared to the WTM. Where the mathematical model suffers is in the prediction of the ectoderm module, with a precision of 50-71%, a recall of 50-67% and a FPR of 45-100%. As above, we use the distance to summarize the findings: the distances of the MIM’s PR and ROC points range from −0.24 to 0.15. We point out that negative distances reflect worse than random predictions. Notably, the single false positive edge prediction in the WTM is also in the ectoderm module, with 71-78% precision, 12-28% recall and 4-17% FPR, with distances between −0.07 and 0.08. The false positives present in both models may be indicative of features of the ectoderm module - such as complexity - that uncover the limitations of our modeling strategy. Alternatively, the false positives could suggest genetic redundancy in this module. For example, redundancy in the network would lead to under-prediction of regulatory edges based on single gene perturbation experiments, which are the primary source of data for the GSN.

Now we compare specific features of the MIM to the GSN. One notable difference between the MIM and the GSN is in the predictions for *tbx-8* and *tbx-9*. In the GSN, these initiation genes are indistinguishable, which is not the case in the MIM. For example, the MIM suggests that *tbx-8* interacts with *lin-26, hlh-1,* and *nob-1*, whereas there is no such prediction for *tbx-9*. Furthermore, of these two initiation genes, only *tbx-9* is found to regulate *elt-3* in the MIM, while in the GSN both regulate *elt-3*. On the chromosome, *tbx-8* and *tbx-9* are adjacent genes with a shared upstream region (they are on opposite strands of the DNA), and this genomic organization has suggested that they are co-regulated [22]. However, because the MIM is built from gene expression data, these model differences reflect differences in the observed behavior of the *tbx-8* and *tbx-9* transcripts, and suggest that the regulation of these genes may be more complicated than previously thought.

An important feature of the MIM is the involvement of *pal-1* not just as a key regulator of other genes, but also as a participant in feedback loops commonly found in gene regulatory networks. Both the GSN and the MIM identify *tbx-8* and *tbx-9* as regulators of *pal-1*, and include the two-cycles *pal-1 ← → tbx-8/tbx-9*. The mathematical model additionally suggests non-initiation genes as regulators of *pal-1*, namely, *hlh-1nob-1*, and *cwn-1*. The genes *pal-1 → nob-1 → cwn-1* form a feedforward loop (with each gene also providing feedback on *pal-1*), while *pal-1 → scrt-1 → hlh-1* and *pal-1 →* (*nhr-25lin-26*) *→ nob-1* form feedback loops. (Note that in a feedforward loop the first element goes to the last, whereas in a feedback loop the last goes to the first.) Other feedforward loops shared by the GSN and the MIM are *pal-1 → hnd-1 → lin-26* and *pal-1 → lin-26 → hlh-1*. Since *pal-1* is both provided maternally and transcribed zygotically, feedback loops in the MIM identify potential zygotic activities for *pal-1*. While other studies have demonstrated the general importance of zygotic *pal-1*[23-25], the ability to separate prospective zygotic from maternal roles is a unique features of the MIM compared to the other models.

The MIM identifies possible alternative network architectures compared to the GSN. Both the GSN and MIM predict the directed paths *lin-26 → nhr-25 → elt-1* and *hnd-1 → lin-26 → hlh-1*, the latter suggesting an alternate path of the direct regulation of *hlh-1* by *hnd-1* represented in the GSN. Another example of an alternative path is that from *scrt-1* to *vab-7*, shown to be direct in the GSN; however, another explanation is that *scrt-1* acts on *vab-7* through *hnd-1*, as suggested by the MIM. In building the GSN, we assumed that experimentally confirmed edges were direct. However, we recognize that gene expression changes might result from either direct or indirect effects. The MIM suggests cases where alternative (indirect) regulatory relationships might explain the observed data.

A comparison of the non-interactions between the MIM and the GSN could not be performed. While such information can in principle be extracted from the chosen modeling methods, there were no non-interactions being reported by MSA and adjusting the threshold for COV resulted in all genes not interacting. As there were no conclusive results, we exclude comparison of non-interactions involving the MIM.

We evaluated the MIM for the same graph theory metrics as we used for the GSN. The average path length is about 3, and therefore less than 4. The nodes with an out-degree greater than half of the network are *pal-1* and *nhr-25*. The average in-degree of the nodes is 3, and - compared to the GSN - the MIM exhibits a narrower in-degree range (2–6). No node exhibits an in-degree greater than half of the network, but the nodes with greatest in-degree are *pal-1* (indeg = 6), *nhr-25* (indeg = 5), and *nob-1* (indeg = 5). We conclude that the MIM derived from wild-type data exhibit similar network features to those of other gene regulatory networks.

Altogether, we have mathematically reverse engineered a model based on wild-type gene expression data. We employed a pipeline modeling strategy that benefits from the unique strengths of two distinct modeling methods, and show that the order of method in the pipeline does not have a big impact on outcome. The model is enriched for positive predictions compared to random guesses, as well as compared to a knowledge-driven model built from the same data. A key benefit of the model is that it extracts information and offers predictions beyond the focus of the original experimental framework. We conclude that complementing knowledge-driven insight with systematic modeling approaches has the potential to improve predictability, prioritize future experiments, and suggest new network features compared to reliance only on knowledge-driven models.

## Discussion

### Mathematical modeling and experimental data sets

We have developed a Mathematically Inferred Model (MIM) for the gene regulatory network underlying development of the C cell lineage of *C. elegans* using gene expression time course data recovered from wild-type animals. We have compared this model to a Gold Standard Network (GSN) built from the data of gene perturbation experiments, and found that the MIM predicts gene interactions better than chance, and extracts a larger and richer set of gene interactions than a knowledge-driven biological model (the Wild Type Model (WTM)) produced from the same wild-type data set. In fact 65% of the MIM’s predictions are validated by at least two sources of experimental evidence. Overall, we conclude that mathematical models of this type can complement experimenter insight to suggest unexpected regulatory relationships and to guide prioritization of future experiments.

One of the main contributions of this work is to demonstrate to predictive power of data-driven models. An important consequence is that it broadens the potential data sets and methods for modeling of biological networks. We used experimental data that were produced to discover new genes involved in *C. elegans* C cell development, but they were not specifically collected for model production [13]. Indeed, the genes included in the network were not selected prior to data collection, but rather were an outcome of the original experiment. This argues that experiments that are well-designed to address specific biological questions can offer data directly useful for modeling. From the modeling perspective, we applied a modeling method (the Minimal Sets Algorithm (MSA)) that has been applied previously to data sets that include perturbation experiments [26-29]. This work suggests that, at least when applied in combination with other modeling methods, MSA can extract meaningful information from wild-type-only data sets. Finally, the model demonstrates that pipeline modeling approaches can be applied to large, experimentally-derived data sets. Altogether, our results provide an example of the utility of mathematically-assisted analysis of experimental data, whether or not the data were originally collected within a modeling framework.

### Modeling insights to *C. elegans* development

This work produced network models of two distinct types: a knowledge-driven network based on a systematic annotation of experimental evidence (the GSN) and a data-driven model that utilizes mathematical modeling methods to infer network features (the MIM). Both methods provide an integrated network that evaluates the regulatory relationship within the defined ectoderm and mesoderm sub-modules, but also among all of the network genes. Both networks demonstrate the potential for integration and cross-talk among all of the genes in the C lineage. In particular, the networks incorporate the genes identified as “mixed” (participating in development of both mesoderm and ectoderm) that were not included in the Experimentally Derived Model (EDM) of [14]. This work demonstrates how modeling approaches can suggest additional regulatory relationships beyond the scope of the original questions addressed by the experiments.

Another biological phenomenon highlighted by the networks is functional redundancy. Based on genetic tests, *tbx-8* and *tbx-9* are functionally redundant, in that animals only exhibit embryonic defects when both genes are disrupted or subject to knockdown [22,30]. For this reason, they are grouped together in the WTM and the EDM [13,14]. Nevertheless, gene-specific probes identify notable differences in the expression abundance and behavior of these two genes ([13], this work). These differences result in distinct regulatory relationships for each gene in the MIM. While it is clear that the biological system is sufficiently robust to only exhibit defects when both genes are disrupted, it will be interesting to test whether gene expression differences can be detected in single gene mutants. Similarly, one source of false positives in the MIM could be functional redundancy not uncovered in the experiments used for the GSN. The redundancy between *tbx-8* and *tbx-9* likely reflects direct compensation (based on the sequence similarity of the two genes). Another paralogous gene pair in the GSN is *elt-1* and *elt-3*, and some functions may be shared between them [31]. An alternative possibility is that functional redundancy results from a distributed network architecture or pathway compensation, as is seen among the non-paralogous mesodermal genes *hlh-1hnd-1* and *unc-120*[32]. Knockdown of network genes in combination could identify the potential for within-network redundancy, and characterize whether features of the network architecture are responsible.

## Conclusions

Many models of biological systems are primarily knowledge-driven, and therefore rely on the availability of suitable data with which to build a model. This approach places the burden on experimentalists to produce data suitable for model-building, and - in practice - limits the number of processes that can be modeled. The argument in favor of a knowledge-driven approach is its track record - it is the foundational method used to apply mathematics to the physical sciences. Indeed, this approach has lead to the insights and formalisms that result in physical theories and laws. Unfortunately, many current computational models of biology describe the available data well, but they predict new results, or extend to related but distinct biological processes, with limited accuracy. Therefore, unlike the powerful ability of mathematics to describe, unify, and predict outcomes in the physical sciences, the promise of mathematical modeling in biology is yet to be realized - a phenomenon that has been described as the “unreasonable ineffectiveness of mathematics in biology” [33]. While we acknowledge the importance knowledge-based models, our current work illustrates the value of data-driven methods to uncover non-intuitive network features, and to use mathematical approaches to guide future experiments based on a preliminary set of descriptive data.

## Methods

### Building the gold standard network

The Gold Standard Network was built based on experimental data curated from the scientific literature. A primary source of data is [14], which includes gene expression analysis in strains systematically perturbed for each gene using RNAi-mediated gene knockdown, and yeast one-hybrid (DNA binding) studies. Both of these data types are directional. Additional gene interaction data were recovered from studies on individual genes, and include gene expression analysis (directional; [12,16,22,34-36]) and synthetic gene interactions (non-directional; [22,30,32,37,38]). In words, network edges were assigned if two or more of the following criteria were met: 1) Yanai *et al.*[14] gene expression change Z score significance was greater than or equal to 2, 2) Yanai *et al.*[14] gene expression fold change was greater than or equal to 2x, 3) Yanai *et al.*[14] yeast one hybrid experiment exhibited a positive result, or 4) a positive relationship was reported in the studies on individual genes (each positive result was counted independently). The rationale for these criteria is to balance our presumption that the alteration of gene expression level in perturbation experiments is the gold standard for demonstrating regulatory relationships with our recognition that DNA binding results support direct interactions, and that other types of experiments (including the replication of results by different research groups) are valuable in the validation of perturbation experiments. Furthermore, we require that each edge satisfies at least two criteria as we aim to produce a gold standard network in which we have a high level of confidence in all of the included edges. This approach may result in a more conservative network, that is a network with fewer edges than if there were fewer criteria to satisfy; however, we expect that our strategy minimizes bias toward better matches with complex models as compared to less complex models. Since knockdown experiments using RNAi (which reduces target gene RNA abundance) are an important source of data for the network, the experimental method precludes detection of prospective self-regulatory relationships. Therefore, the network does not include any auto-regulatory loops. The GSN is provided as a graph in Figure2, with the data in Additional file 1: Table S 1, Tab “GSN”.

The network is represented as a mixed graph, meaning the graph has directed as well as undirected edges. The graph is encoded as an adjacency matrix where an entry (*i, j*) has value

1, for a directed edge, if gene *j* regulates gene *i*

−1, for an undirected edge, if there is an interaction between genes *i* and *j*, but the direction of the interaction is unknown

0, for a non-edge, if there is no interaction between genes *i* and *j*

-, if the interaction between genes *i* and *j* is unknown

The entries of the matrix are set according to the following rules:

1, if (*sig* ≥ 2 and *mag* ≥ 2) or (*sig* ≥ 2 and *y1h* = 1) or (*gen* ≥ 2) or (*sig* ≥ 2 and |*gen*| = 1)

−1, if |*gen*| ≥ 2

0, if *mag* = 0 and *sig* = 0 and *y1h* = 0

-, otherwise

where *sig* denotes the significance of the transcript abundance, *mag* denotes the magnitude (fold change) of the transcript abundance, *y1h* denotes the existence of a yeast one-hybrid interaction, and *gen* denotes the existence of genetic interactions (described above). The values for *sig, mag,* and *y1h* were extracted from [14], and the values for *gen* were extracted from the references listed. A numerical value for *y1h* and *gen* indicates the number of interactions of said type. Here we use | . | to denote the absolute value operator.

### Building the mathematically inferred model

The data for the Mathematically Inferred Model (MIM) are derived from gene expression microarray time-course analysis of RNA from whole embryos carried out in [13]. The RNA abundance levels in wild-type embryos (who have one C cell) and *mex-3(zu155); skn-1(RNAi)* embryos (who have 11 cells that develop like the normal C cell (~11/12 of the embryo)) were utilized to develop the model. We anticipate that in each of these experimental conditions the cellular decisions distinguishing mesodermal from ectodermal cell types within the C cell lineage take place, with perhaps a greater “signal” from the *mex-3(zu155); skn-1(RNAi)* animals due to the reduction of “noise” from other cell lineages. Both of these genotypes are considered “wild type” from the perspective of model-building, as neither disrupts any of the genes within the modeled network, and were used as independent time series. The model (produced from the pipeline MSA-COV) is provided as a graph in Figure4, the data in Additional file 1: Table S 1, Tab “MIM (MS-COV)”. An alternative model (produced from the pipeline COV-MSA) is provided as a graph in Figure 1S in Additional file 2, with the data in Additional file 1: Table S 1, Tab “MIM (COV-MS)”.

The data set used to build the model is the gene transcript abundance measured at 10 developmental time points in [13]. Since we are interested in modeling the network of genes influenced by *pal-1* (as identified in both [13] and [14]), we extracted the time-course data for the following 15 genes: *pal-1, tbx-8, tbx-9, elt-1, lin-26, nhr-25, elt-3, hnd-1, hlh-1, unc-120, scrt-1* (previously labeled C55C2.1)*, vab-7, nob-1, cwn-1,* and *mab-21.* The genes in this listing had unique transcript probes, with the exception of *pal-1*, which had three probes on the microarray; *elt-3*, with two; *nhr-25*, with two; *nob-1*, with three; and *lin-26*, with two. In fact, for those genes with 3 probes, there are two probes that are close to the 5' end of the gene and one close to the 3' end. The data for the probes near the 5' end are more similar than that of the probe near the 3' end. As there was not enough information to assess the central tendency of the data, we did not combine the data for multiple probes for a given gene. We represented each distinct data set with a variable, giving 22 nodes in the network.

The model is represented as a mixed graph, meaning the graph has directed as well as undirected edges. The graph is encoded as an adjacency matrix where an entry (*i, j*) has value

1, for a directed edge, if gene *j* regulates gene *i*

−1, for an undirected edge, if there is an interaction between genes *i* and *j*, but the direction of the interaction is unknown

0, for a non-edge, if there is no interaction between genes *i* and *j*

We used a pipeline approach to build a mathematical model. We combined the following statistical and algebraic methods: covariance (COV), which measures how much variables change together; and the Minimal Sets Algorithm (MSA) [26], which identifies directional functional relationships between genes. Next we provide justification for the choice of methods.

Because each probe for a gene is represented with a variable, dependence among these variables needed to be preserved: modeling methods typically assume independence among variables. Since covariance measures how much variables change together, we combined it with a threshold to couple data that should be considered to be the same. However, covariance alone cannot identify directed interactions. Therefore, covariance needed to be paired with a method that can detect directionality in the inferred relationships. While there are numerous choices [5-8], most inference methods return a few likely models. The Minimal Sets Algorithm is a network inference method that was developed to compute all minimal models from input–output data. The algorithm identifies sets of variables that are most likely responsible for the response given the input data; moreover the sets are *minimal* in the sense they include the fewest variables that reproduce the response. An advantage of MSA is that it allows for a rigorous analysis of the model space since all possible models are constructed; moreover, it does not have any requirements for the input data. As a result, we combined MSA and COV for their suitable and complementary features.

For each method, we constructed an adjacency matrix. Since directionality cannot be deduced from COV, the covariance adjacency matrix, denoted *cov*(*i, j*), has (0, –1) entries where 0 means “no interaction” and −1 means “undirected interaction”. To construct *cov*(*i, j*), we first computed the standard (*n* x *n*)-variance-covariance matrix using MATLAB's built-in cov function [39], where *n* is the number of variables. We then chose a threshold such that the entries of the adjacency matrix corresponding to the probeset of a given gene all have a value of −1. We found that the median across the entire matrix yielded an appropriate threshold.

Since MSA does provide directionality, its adjacency matrix, denoted *msa*(*i, j*), has (0, 1) entries where 0 means “no interaction” and 1 means “directed interaction” from gene *j* to gene *i*. To construct *msa*(*i, j*), we followed the procedure as outlined in [26] which we implemented in the computer algebra system *Macaulay2*[40]. We discretized the wild-type data set using the method of [41]: the data were discretized to 7 states. Then we applied MSA to the discretized data. Since multiple possible models were returned, we discarded models that did not satisfy a “no crosstalk” requirement (ectodermal genes should not directly regulate mesodermal genes, and mesodermal genes should not directly regulate ectodermal genes) wherever possible and used the scoring method (Algorithm 8 with the *S*_1_*T*_1_ score in [26]) to select the most likely such model. This “no crosstalk” rule is included as it was an assumption of the WTM derived from the same data set.

Below we provide two strategies for developing a pipeline using COV and MSA.

*COV-MSA*. One strategy is to choose the undirected edges from the output of COV and then to incorporate directed edges from MSA. For genes *i* and *j*, if both *cov*(*i, j*) = −1 and *msa*(*i, j*) = 1 then we say that there is a directed edge *j → i* in the resulting model. If there is no directed edge in either direction between *i* and *j* in the MSA model and *cov*(*i, j*) = −1, then we say there is an undirected edge between *i* and *j*. For a given gene *i*, if there is no directed self-loop in the MSA model, but the variance of its data is above the chosen threshold (that is, *cov*(*i, i*) = −1), we discard such edges as self regulation cannot be determined from covariance. For genes with multiple probes, an entry is set to 1 (or −1) if all of its probes have a value of 1 (or −1) according to the rules below. This results in an adjacency matrix for a 15-node network, as desired. In summary, the two adjacency matrices are combined using the following rules:

1, if *cov*(*i, j*) = −1 and *msa*(*i, j*) = 1

−1, if *i ≠ j* and *cov*(*i, j*) = −1 and *msa*(*i, j*) = 0 and *msa*(*j, i*) = 0

0, if *cov*(*i, j*) = −1 and *msa*(*i, j*) = 0 and *msa*(*j, i*) = 1 or if *cov*(*i, j*) = 0.

*MSA-COV*. Another strategy is to choose the directed edges from the output of MSA and then to incorporate undirected edges from COV. We include an undirected edge between two distinct genes *i* and *j* if there is no directed edge in either direction between them and *cov*(*i, j*) = −1. As with COV-MSA, undirected self-loops are discarded and genes with multiple probes were handled as described above. A summary of the algorithm follows:

1, if *msa*(*i, j*) = 1

−1, if *i ≠ j* and *cov*(*i, j*) = −1 and *msa*(*i, j*) = 0 and *msa*(*j, i*) = 0

0, otherwise.

### Building the wild type model

The Wild Type Model (WTM) is based on the model offered in Baugh *et al.* ([13]; modified from their Figure 9) as an interpretation of their wild-type gene transcript abundance time course data. It is a knowledge-driven biological model for comparison to the Mathematically Inferred Model built from the same source data set. This model emphasizes the temporal and spatial relationship of expression for each gene. In words, each directional edge included in Baugh *et al.* (Figure 9 of [13]) was assigned as a directional edge in the model. Genes assigned to a particular time phase might influence genes in that time phase or later, but were restricted (blocked) from influencing genes in an earlier time phase. Furthermore, transcription factor genes assigned to a particular tissue type (mesodermal or ectodermal) were restricted from influencing transcription factor genes in the alternative tissue type (a “no cross-talk” constraint also included in the MIM).

The model is represented as a directed graph, meaning the graph has only directed edges. The graph is encoded as an adjacency matrix where an entry (*i, j*) has value

1, for a directed edge, if gene *j* regulates gene *i*

0, for a non-edge, if there is no interaction between genes *i* and *j*

-, if the interaction between genes *i* and *j* is unknown

The model was constructed directly from Figure 9 of [13]. The WTM is provided as a graph in Figure5, with the data in Additional file 1: Table S 1, Tab “WTM”.

### Comparison of the models to the gold standard network

We assessed the predictability of the models on the interactions observed in the mutant experiments by comparing the models to the Gold Standard Network. We focused on three aspects of the network: the overall structure, the targets of the PAL-1 protein, the structure of the mesoderm and ectoderm modules. We used precision-recall and receiver-operator plots to measure the models’ performance.

Let *TP* denote true positives; *TN*, true negatives; *FP*, false positives; and *FN*, false negatives. A precision-recall (PR) graph is a plot of the precision against the recall. Precision, or positive predictive value (*PPV*), is a measure of how many of the predicted interactions are correct and is defined as $\frac{TP}{TP+FP}$. Recall, or true positive rate (*TPR*), is a measure of how many of the observed interactions are correctly predicted and is defined as $\frac{TP}{TP+FN}$. A receiver-operator curve (ROC) graph is a plot of the true positive rate (which is the same as recall) against the false positive rate (*FPR*). *FPR* is a measure of how many of the known non-interactions are incorrectly predicted as interactions and is defined as $\frac{FP}{FP+TN}$.

In PR space, a classifier has strong predictive value if its points lie in the upper, right-hand corner of the graph, representing a precision and recall close to 1. In ROC space, a classifier has strong predictive value if its points lie in the upper, left-hand corner of the graph, representing an *FPR* close to 0 and a *TPR* close to 1. In either space, points that lie on the dashed line are considered to be no better than random guesses and points below the line are considered to be weak classifiers. Even though it is known that classifiers that dominate in one space will dominate in the other space (meaning one can go back and forth between PR and ROC curves), we show both plots here to provide a more complete representation of the assessment [42]. Note that recall is the same as *TPR*; we will use these words interchangeably. We employ both terms here to be consistent with the standard classifier nomenclature.

We provide the justification for the computation of the aforementioned quantities below. Since we are comparing adjacency matrices of mixed graphs, we had to modify the formula of the precision and recall to account for half-right predictions; that is, an undirected edge in the model for which the corresponding edge in the GSN is directed. Let *HR* denote half right, *mim*(*i, j*) refer to the adjacency matrix for the mathematical model, and *gsn*(*i, j*) refer to the adjacency matrix for the Gold Standard Network. We defined the following:

*TP* := (*mim*(*i, j*) = *gsn*(*i, j*) = 1) or (*mim*(*i, j*) = 1 and *gsn*(*i, j*) = −1)

*TN* := *mim*(*i, j*) = *gsn*(*i, j*) = 0

*HR* := *mim*(*i, j*) = −1 and *gsn*(*i, j*) = 1

*FP* := *mim*(*i, j*) = 1 and *gsn*(*i, j*) = 0

*FN* := *mim*(*i, j*) = 0 and *gsn*(*i, j*) = 1 or −1

Then we modified the standard formulas as follows:

(1) $$TPR:=\frac{TP+0\text{.5HR}}{\left(TP+0\text{.5HR}\right)+\left(FN+0\text{.5HR}\right)}$$

(2) $$PPV:=\frac{TP+0\text{.5HR}}{\left(TP+0\text{.5HR}\right)+FP}\text{.}$$

For a measure of the total number of predictions, we compute *TP + TN + HR + FP*; for the total number of predictions that are correct, we use *TP + TN +* 0.5*HR*.

The results of the analyses are provided in the top row of Figure6 and the accompanying table is in Additional file 1: Table S 1, Tab “Overall”.

To evaluate each pipeline, we computed the distance of the points in the PR and ROC plots from the appropriate diagonal line; see below for definitions of these plots. In ROC space, the distance of a point (*x,y*) from the diagonal line *y* = *x* is measured as the length of difference of the vector (*x,y*) and the projection of (*x,y*) onto the vector (1,1); that is, the distance is given by

(3) $$\sqrt{{\left(x-\frac{x+y}{2}\right)}^{2}+{\left(y-\frac{x+y}{2}\right)}^{2}}=\frac{\sqrt{2}\left(x-y\right)}{2}\text{.}$$

In PR space, the distance of a point (*x,y*) from the diagonal *y* = 1 – *x* is measured as the length of difference of the vector (*x–*1*,y*) and the projection of (*x–*1*,y*) onto the vector (−1,1); that is, the distance is given by

(4) $$\sqrt{{\left(\left(x-1\right)-\frac{\left(x-1\right)-y}{2}\right)}^{2}+{\left(y+\frac{\left(x-1\right)-y}{2}\right)}^{2}}=\frac{\sqrt{2}\left(x-1\right)+y}{2}\text{.}$$

For points below the diagonal, we assigned them with a negative distance, realized by the above expressions on the right, to reflect their position with respect to the line of random guesses. To determine the best pipeline approach, we added all distances (positive and negative) in both spaces and the pipeline with the largest positive distance from random is deemed the best (see Additional file 1: Table S 1, Tab “Overall”).

Since we have only one PR or ROC point per predicted feature (ectoderm modules, mesoderm modules, targets of PAL-1, etc.), we do not have a curve as is typically expected in these types of analyses. Hence we cannot use the standard Area Under the Curve (AUC) to measure the overall performance of the models. We constructed the distance measure defined above to be a one-dimensional variant of the AUC. The greatest distance possible of any point from the line of random guesses is 1/$\sqrt{2}\text{,}$ which is approximately 0.7. Furthermore, we use the total distance, that is the sum of the distances over each measured feature and over both PR and ROC plots, as a comprehensive indicator of performance. The maximum value of the total distance is 12/$\sqrt{2}\text{,}$ which is approximately 8.49.

The model using the pipeline COV-MSA has a total distance of 2.3, whereas the model from MSA-COV has a total distance of 2.8. Plots of the distances for both PR and ROC graphs are provided in the bottom row of Figure6; the accompanying table is in Additional file 1: Table S 1, Tab “Overall”.

## Competing interests

The authors declare that they have no competing interests.

## Authors' contributions

BS and HMC designed the modeling strategy, collected data from the literature, and wrote the manuscript. BS formulated the models and performed the analyses. Both authors read and approved the final manuscript.

## Supplementary Material

Additional file 1 ** Table S1. Supporting Excel workbook for modeling and analysis.** All presented models, computations, and results are supported by the Excel workbook in Additional file 2; note that many of the spreadsheets in the workbook contain formulas that reference multiple spreadsheets. See the tab “README” for a listing and description of the contents. Click here for file

Additional file 2 **Graphs and performance plots for the MIM built using COV-MSA. Figure S1.** contains the graphs comprising the MIM using the COV-MSA pipeline. S2 contains the Precision-Recall and ROC plots for this model. Click here for file
