# Supplementary material for: A regulatory network modeled from wild-type gene expression data guides functional predictions in Caenorhabditis elegans development
Source: BMC Syst Biol. 2012 Jun 26;6:77. doi: 10.1186/1752-0509-6-77 (PMC3463499; doi:10.1186/1752-0509-6-77)
Supplement: Additional file 2 — Graphs and performance plots for the MIM built using COV-MSA. Figure S1. contains the graphs comprising the MIM using the COV-MSA pipeline. S2 contains the Precision-Recall and ROC plots for this model. [file 1752-0509-6-77-S2.pdf]

Additional File 1

Below are the graphs and performance plots for the alternate model MIM built using COV-MSA.

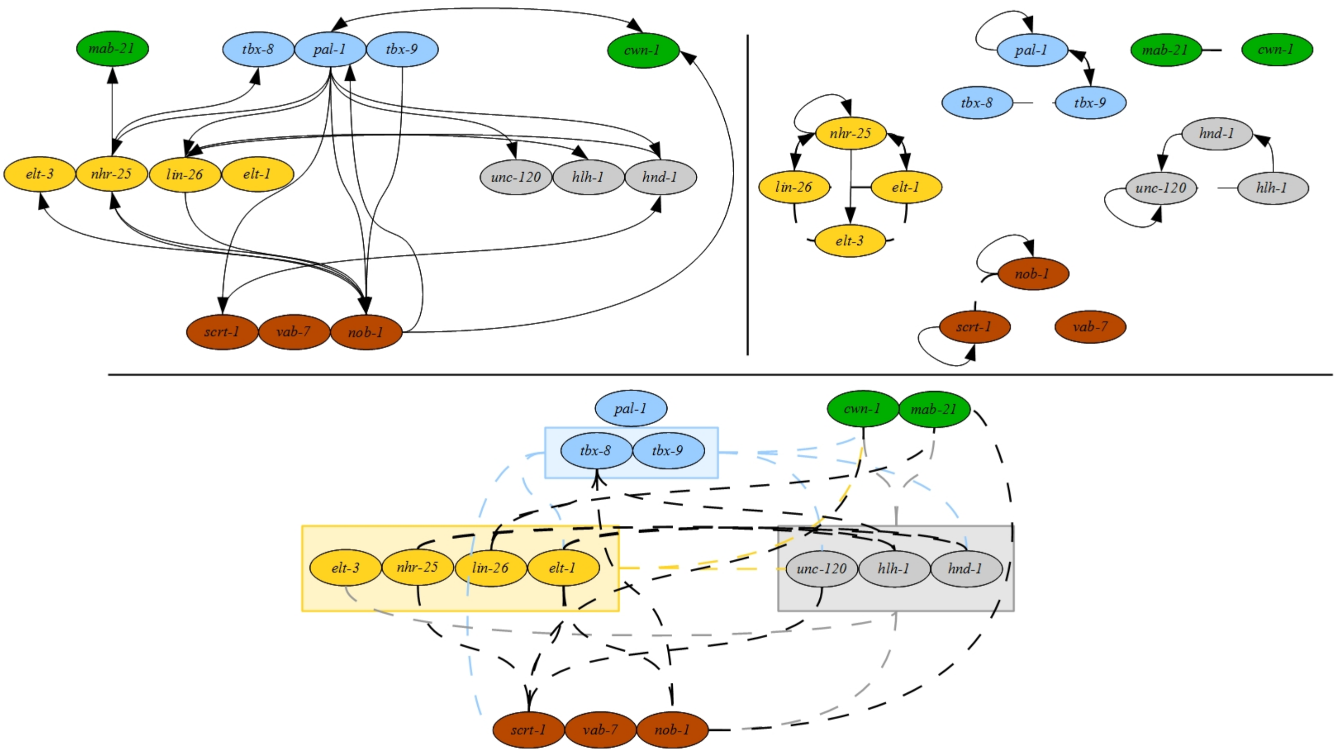

Figure 1S – The Mathematically Inferred Model (MIM) constructed using COV-MSA

See the legend of Figure 4 for a full key.

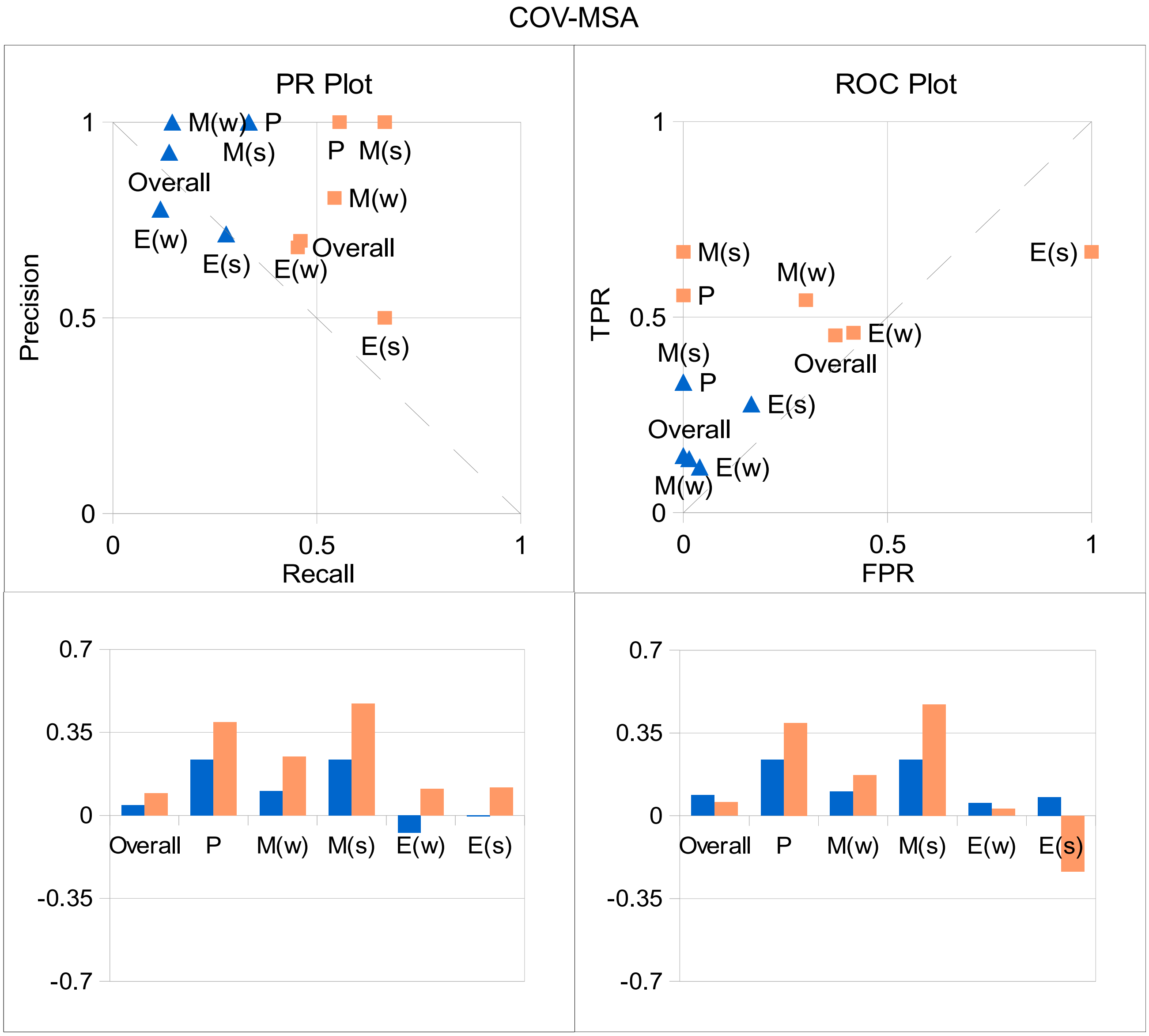

Figure 2S – Performance of the MIM using COV-MSA

In the upper left is a precision-recall (PR) plot and in the upper right is a receiver operator characteristic (ROC) plot. Blue triangles and orange squares represent data points for the WTM and the MIM, respectively. In the bottom row are the distances of the points in the upper plots from the dashed lines. See the legend of Figure 6 for a full key of terms.
